# Exploring the association of organochlorine pesticides exposure and hearing impairment in United States adults

**DOI:** 10.1038/s41598-022-15892-2

**Published:** 2022-07-13

**Authors:** Lili Long, Xinghua Tang

**Affiliations:** 1 Department of Otorhinolaryngology, Sichuan University Hospital of Sichuan University, Chengdu, 610065 Sichuan China; 2 Department of Otorhinolaryngology Head and Neck Surgery, Sichuan Provincial People’s Hospital, University of Electronic Science and Technology of China, No. 32, West Section 2, Yihuan Road, Chengdu, 610072 Sichuan China

**Keywords:** Environmental impact, Auditory system, Risk factors

## Abstract

Hearing loss (HL) is a highly prevalent public health concern. Organochlorine pesticides (OCPs) are widely used environmental pollutants harmful to human health. Studies investigating the effects of OCPs exposure on the auditory system in the general population are rare. To explore the association between OCPs exposure and HL in adults, 366 adults aged 20–69 years who participated in the National Health and Nutrition Examination Survey (NHANES, 2003–2004) were investigated. HL was defined as a pure-tone average (PTA) ≥ 20 dB in the better ear. Multivariate linear and logistic regression analyses were conducted to evaluate the association of four selected serum OCPs with PTAs and the risk of HL. In participants aged < 60 years, hexachlorobenzene (HCB) and dichlorodiphenyldichloroethylene (p, p'-DDE) exposure was positively associated with low- and speech-frequency PTAs, and with low-frequency HL, respectively. Risk of HL increased in the highest tertile compared with the lowest tertile of serum HCB and p, p'-DDE (odds ratio [OR]: 4.38, 95% confidence interval [CI]: 0.97–19.80; OR: 16.66, 95% CI: 2.64–105.09, respectively). In this study of US adults aged < 60 years, HCB and p, p'-DDE exposure was positively associated with HL. HCB and p, p'-DDE may be potential risk factors for HL.

## Introduction

Hearing loss (HL) is a highly prevalent sensory disorder. Globally, it is the third most common cause of disability in humans. It affects people of all ages, causes enormous financial burden, and afflicts people through the loss of education, communication, and social interaction when HL is left unaddressed. Over 1.5 billion people currently have HL, and this number could grow to 2.5 billion by 2050^1^. To prevent and treat HL, several studies have been conducted to investigate its common causes, such as noise exposure, aging, and ototoxic drug use. Concerns have also been raised regarding the role of exposure to environmental pollutants in the development of HL^2^.

Organochlorine pesticides (OCPs) are persistent organic environmental pollutants that bioaccumulate in food chains. Although some OCPs have been banned or restricted for decades, their extensive usage, long half-lives, and bioaccumulation still affect human health^3^. Previous studies have shown that OCPs may cause immune dysfunction, endocrine disruption, neurobehavioral and cognitive impairment, and may have chronic effects on reproductive potential, as carcinogens^4–8^.

Recently, interest has increased regarding the effect of environmental pollutants on the development of hearing impairment^2^. Although animal studies, experimental studies on humans, and epidemiological surveys have indicated the toxicity of polychlorinated biphenyls (PCBs), another important organochlorine pollutant, on the auditory system, studies investigating the effects of exposure to OCPs on the auditory system are limited^9–13^. Animal studies have addressed the ototoxicity of organochlorine pesticides dichlorodiphenyltrichloroethane (DDT) and hexachlorobenzene (HCB) in rats^14–16^. A previous study reported that exposure to OCPs, including HCB, β-hexachlorocyclohexane (β-HCH), and p, p'-dichlorodiphenyldichloroethylene (p, p'-DDE, a metabolite of DDT), at environmental concentrations during infancy, may be associated with hearing impairment^17^. A case–control study indicated that exposure to α-hexachlorocyclohexane (α-HCH), an OCP, might be a potential risk factor for HL^18^. In this study, we investigated whether there were associations between environmental exposure to OCPs and HL in adults who participated in the National Health and Nutrition Examination Survey (NHANES) 2003–2004. To our knowledge, this is the first cross-sectional study to investigate the effect of OCPs exposure on the auditory system in the general adult population in the US.

## Results

### Baseline characteristics of study participants

Table 1 shows the baseline characteristics of the participants in this study (n = 366) aged between 20 and 69 years, including 195 females (weighted mean, 40.74 ± 11.77 years) and 171 males (weighted mean, 41.52 ± 14.07 years). The mean ± standard deviation (SD) values of low-, speech-, and high-frequency pure-tone average (PTA) hearing thresholds in the male participants were 8.34 ± 6.76 dB, 11.81 ± 8.33 dB, and 22.84 ± 17.30 dB, respectively. The hearing thresholds were 8.86 ± 7.87 dB, 9.54 ± 7.99 dB, and 15.48 ± 13.90 dB in the female participants, respectively. Overall, HL rates were 14.59% and 8.63% among the male and female participants, respectively. There were statistically significant differences between the male and female participants in speech- and high-frequency PTA, serum cotinine level, and loud noise/music exposure (all *P* < 0.05).

**Table 1:** The weighted demographic characteristics of study participants.

Variables	Male (N = 171)	Female (N = 195)	*P* value^a^
**Continuous variables, mean ± SD**			
Age (years)	41.52 ± 14.07	40.74 ± 11.77	0.5654
BMI (kg/m^2^)	28.23 ± 5.13	28.38 ± 6.74	0.8126
Low-frequency PTA (dB)^b^	8.34 ± 6.76	8.86 ± 7.87	0.5000
Speech-frequency PTA (dB)^b^	11.81 ± 8.33	9.54 ± 7.99	**0.0084**
High-frequency PTA (dB)^b^	22.84 ± 17.30	15.48 ± 13.90	**< 0.0001**
**Categorical variables, %**			
Race/Ethnicity			0.5970
Mexican American	9.81	7.23	
Non-Hispanic White	69.76	70.27	
Non-Hispanic Black	12.82	11.59	
Other races	7.61	10.90	
Education level			0.3444
Below high school	15.64	11.20	
High school	26.24	24.17	
Above high school	58.12	64.63	
BMI (categorical)			0.0911
Underweight (< 18.5 kg/m^2^)	0.76	2.89	
Normal (≥ 18.5 kg/m^2^, < 25 kg/m^2^)	26.79	33.38	
Overweight (≥ 25 kg/m^2^, < 30 kg/m^2^)	37.94	27.33	
Obesity (≥ 30 kg/m^2^)	32.66	35.75	
Not recorded	1.86	0.65	
Diabetes	6.50	5.78	0.7748
Hypertension	19.15	26.40	0.0891
Serum cotinine (≥ 10 ng/ml)	45.65	33.07	**0.0138**
Firearm noise exposure	11.11	3.68	**0.0058**
Loud noise/music exposure	37.16	18.58	**< 0.0001**
Hearing loss^c^	14.59	8.63	0.0737

*BMI* body mass index, *PTA* pure-tone average.

^a^*P* values of continuous variables and categorical variables were calculated by weighted linear regression model and weighted chi-square test, respectively.

^b^Low-, speech-, high-frequency PTA values in the better ear were computed from the average of hearing thresholds of 0.5, 1 and 2 kHz, 0.5, 1, 2 and 4 kHz, 4, 6 and 8 kHz, respectively.

^c^Hearing loss was defined as the PTA value ≥ 20 dB in the better ear.

Significant values are in bold.

### Comparison of variables among low-, speech- and high-frequency HL groups by univariate analysis

The univariate analysis (Table 2) showed that gender and firearm noise exposure had significant correlations with speech-frequency PTA; gender, race (non-Hispanic Black vs. Mexican American), and serum cotinine level had significant correlations with high-frequency PTA; age, education level (above high school vs. below high school), diabetes, hypertension, and the four OCPs were significantly correlated with low-, speech-, and high-frequency PTA (all *P* < 0.05).

**Table 2:** The univariate analysis of comparison of variables in hearing loss group.

Variables	N (%)/Mean ± SD	Low-frequency HL (N = 31)	Speech-frequency HL (N = 52)	High-frequency HL (N = 132)
		OR (95% CI) *P* value	OR (95% CI) *P* value	OR (95% CI) *P* value
Age	41.74 ± 13.91	**1.10 (1.06, 1.14) < 0.0001**	**1.13 (1.09, 1.17) < 0.0001**	**1.12 (1.09, 1.14) < 0.0001**
Gender (female)	195 (53.28%)	0.93 (0.45, 1.94) 0.8460	**0.45 (0.25, 0.83) 0.0102**	**0.37 (0.24, 0.58) < 0.0001**
**Race/Ethnicity**				
Mexican American	77 (21.04%)	1.0	1.0	1.0
Non-Hispanic White	178 (48.63%)	0.70 (0.29, 1.67) 0.4148	0.74 (0.37, 1.48) 0.3952	1.23 (0.71, 2.13) 0.4536
Non-Hispanic Black	80 (21.86%)	0.40 (0.12, 1.35) 0.1392	0.40 (0.15, 1.03) 0.0585	**0.48 (0.24, 0.97) 0.0397**
Other races	31 (8.47%)	0.81 (0.20, 3.21) 0.7639	0.44 (0.12, 1.65) 0.2256	0.68 (0.27, 1.67) 0.3970
**Education level**				
Below high school	77 (21.04%)	1.0	1.0	1.0
High school	90 (24.59%)	0.68 (0.28, 1.67) 0.3962	0.60 (0.28, 1.31) 0.2031	0.60 (0.32, 1.11) 0.1031
Above high school	199 (54.37%)	**0.26 (0.10, 0.64) 0.0034**	**0.37 (0.18, 0.74) 0.0050**	**0.50 (0.29, 0.86) 0.0117**
**BMI (kg/m**^**2**^**)**				
Underweight (< 18.5)	4 (1.09%)	1.0	1.0	1.0
Normal (≥ 18.5, < 25)	103 (28.14%)	0.25 (0.02, 2.72) 0.2563	0.29 (0.03, 3.06) 0.3011	1.18 (0.12, 11.77) 0.8905
Overweight (≥ 25, < 30)	124 (33.88%)	0.35 (0.03, 3.63) 0.3800	0.58 (0.06, 5.83) 0.6412	2.03 (0.20, 20.05) 0.5456
Obesity (≥ 30)	128 (34.97%)	0.23 (0.02, 2.41) 0.2185	0.62 (0.06, 6.27) 0.6876	1.86 (0.19, 18.40) 0.5953
Diabetes	28 (7.65%)	**5.48 (2.18, 13.78) 0.0003**	**4.69 (2.05, 10.70) 0.0002**	**4.20 (1.84, 9.59) 0.0006**
Hypertension	89 (24.32%)	**3.28 (1.55, 6.95) 0.0019**	**4.35 (2.36, 8.02) < 0.0001**	**2.54 (1.56, 4.15) 0.0002**
Serum cotinine (≥ 10 ng/ml)	122 (33.33%)	1.29 (0.61, 2.76) 0.5078	1.30 (0.71, 2.38) 0.3979	**1.60 (1.03, 2.51) 0.0383**
Firearm noise exposure	20 (5.46%)	2.00 (0.55, 7.26) 0.2896	**2.80 (1.02, 7.64) 0.0451**	2.27 (0.92, 5.64) 0.0764
Loud noise/music exposure	94 (25.68%)	1.42 (0.64, 3.14) 0.3831	1.66 (0.89, 3.10) 0.1140	1.01 (0.62, 1.64) 0.9804
Log HCB (ng/g lipid)	3.89 ± 0.63	**1.70 (1.02, 2.82) 0.0401**	**1.92 (1.24, 2.96) 0.0034**	**1.51 (1.06, 2.14) 0.0215**
Log p, p'-DDE (ng/g lipid)	8.01 ± 1.60	**1.65 (1.31, 2.09) < 0.0001**	**1.58 (1.30, 1.91) < 0.0001**	**1.41 (1.22, 1.63) < 0.0001**
Log nonachlor (ng/g lipid)	3.83 ± 1.31	**1.90 (1.39, 2.60) < 0.0001**	**2.14 (1.63, 2.80) < 0.0001**	**2.15 (1.74, 2.66) < 0.0001**
Log dieldrin (ng/g lipid)	2.63 ± 0.90	**1.97 (1.32, 2.95) 0.0009**	**2.11 (1.50, 2.96) < 0.0001**	**2.28 (1.72, 3.01) < 0.0001**

*SD* standard deviation, *HL* hearing loss, *OR* odds ratio, *CI* confidence interval, *BMI* body mass index, *HCB* hexachlorobenzene, *p, p'-DDE* p, p'-dichlorodiphenyldichloroethylene.

Significant values are in bold.

### Multivariate regression analysis: association of OCPs with hearing thresholds and HL

In Supplementary Tables S1 and S2, we estimated the association of the four OCPs with low-, speech-, high-frequency hearing thresholds, and with HL using multivariate linear and logistic regression models, respectively. The covariates included in this analysis were age, sex, race, education level, body mass index (BMI) (categorical), diabetes, hypertension, serum cotinine level, firearm noise exposure, and loud noise/music exposure. All four OCPs were converted to categorical variables (tertiles) and were used as continuous variables to calculate the linear trend. In the unadjusted model (crude model), the *P* value for trend showed that almost all the four OCPs were significantly associated with low-, speech-, and high-frequency PTA hearing threshold shifts and HL. Only the association of HCB with low- and high-frequency HL was not significant. In all the adjusted models, a significant *P* for trend was not observed among the tertiles of the four OCPs and either hearing threshold shifts or HL (all *P* ≥ 0.05).

### Multivariate regression analysis stratified by age: association of OCPs exposure with hearing thresholds and HL

As shown in Tables 3 and 4, the participants were divided into two groups: adults aged < 60 years (N = 308) and adults aged ≥ 60 years (N = 58). HCB exposure was positively associated with low- and speech-frequency PTAs among participants aged < 60 years, when comparing the highest tertile of HCB exposure level with the lowest tertile (β = 1.90, 95% confidence interval [CI]: 0.11–3.70, *P*_trend_ = 0.0354, *P*_interaction_ = 0.0063 in the low-frequency PTA group; β = 1.88, 95% CI: 0–3.77, *P*_trend_ = 0.0454, *P*_interaction_ = 0.0397 in the speech-frequency PTA group). p, p'-DDE showed a similar association (β = 2.84, 95% CI: 0.86–4.81, *P*_trend_ = 0.0054, *P*_interaction_ = 0.0063 in the low-frequency PTA group; β = 3.44, 95% CI: 1.38–5.51, *P*_trend_ = 0.0015, *P*_interaction_ = 0.0448 in the speech-frequency PTA group). However, there was no clear association between HCB or p, p'-DDE exposure and hearing threshold shifts among individuals aged ≥ 60 years (Table 3). Among individuals aged < 60 years old, those with high HCB exposure showed a higher risk of HL than those with low HCB exposure; however, this association was not observed among individuals aged ≥ 60 years (odds ratio [OR]: 4.38, 95% CI: 0.97–19.80, *P*_trend_ = 0.0475, *P*_interaction_ = 0.0101) (Table 4). An increase in the risk of HL was observed in individuals aged < 60 years with p, p'-DDE exposure levels in the highest tertile, compared with those in the lowest tertile (OR: 16.66, 95% CI: 2.64–105.09, *P*_trend_ = 0.0015, *P*_interaction_ = 0.0288); the 95% CI associated with the OR had a wide range. Dieldrin exposure had no statistically significant interactions with age in the prediction of either hearing threshold shifts or HL (*P*_interaction_ > 0.05) (Tables 3 and 4).

**Table 3:** Adjusted associations between OCPs and hearing threshold shifts stratified by age (N = 366): age < 60 group (N = 308) and age ≥ 60 group (N = 58).

			Log HCB (ng/g lipid)			*P*_trend_	*P*_interaction_
			Tertile 1	Tertile 2	Tertile 3		
Low-frequency PTA	Age < 60	Adjusted βs	Reference	1.25 (− 0.52, 3.01)	1.90 (0.11, 3.70)	**0.0354**	**0.0063**
	Age ≥ 60	Adjusted βs	Reference	− 1.06 (− 8.08, 5.95)	− 0.99 (− 8.12, 6.14)	0.8200	
Speech-frequency PTA	Age < 60	Adjusted βs	Reference	1.45 (− 0.40, 3.30)	1.88 (− 0.00, 3.77)	**0.0454**	**0.0397**
	Age ≥ 60	Adjusted βs	Reference	0.77 (− 6.76, 8.29)	0.00 (− 7.65, 7.65)	0.9443	
High-frequency PTA	Age < 60	Adjusted βs	Reference	1.04 (− 2.37, 4.45)	0.54 (− 2.93, 4.01)	0.7321	0.3340
	Age ≥ 60	Adjusted βs	Reference	8.29 (− 6.21, 22.80)	6.84 (− 7.91, 21.60)	0.4813	

			Log p, p'-DDE (ng/g lipid)			*P*_trend_	*P*_interaction_
			Tertile 1	Tertile 2	Tertile 3		
Low-frequency PTA	Age < 60	Adjusted βs	Reference	1.21 (− 0.51, 2.94)	2.84 (0.86, 4.81)	**0.0054**	**0.0063**
	Age ≥ 60	Adjusted βs	Reference	1.21 (− 7.19, 9.62)	− 4.58 (− 12.54, 3.38)	0.0692	
Speech-frequency PTA	Age < 60	Adjusted βs	Reference	1.10 (− 0.71, 2.90)	3.44 (1.38, 5.51)	**0.0015**	**0.0448**
	Age ≥ 60	Adjusted βs	Reference	0.99 (− 8.25, 10.24)	− 3.66 (− 12.42, 5.11)	0.1791	
High-frequency PTA	Age < 60	Adjusted βs	Reference	2.19 (− 1.15, 5.52)	4.43 (0.62, 8.24)	0.0224	0.8135
	Age ≥ 60	Adjusted βs	Reference	1.25 (− 17.34, 19.83)	− 1.63 (− 19.23, 15.98)	0.7144	

			Log dieldrin (ng/g lipid)			*P*_trend_	*P*_interaction_
			Tertile 1	Tertile 2	Tertile 3		
Low-frequency PTA	Age < 60	Adjusted βs	Reference	2.17 (0.41, 3.93)	1.82 (− 0.13, 3.77)	0.0508	0.0961
	Age ≥ 60	Adjusted βs	Reference	3.17 (− 5.98, 12.32)	2.17 (− 6.36, 10.69)	0.8070	
Speech-frequency PTA	Age < 60	Adjusted βs	Reference	1.82 (− 0.02, 3.67)	2.07 (0.02, 4.12)	0.0403	0.3972
	Age ≥ 60	Adjusted βs	Reference	3.75 (− 6.03, 13.52)	4.23 (− 4.87, 13.34)	0.4311	
High-frequency PTA	Age < 60	Adjusted βs	Reference	2.32 (− 1.05, 5.69)	4.98 (1.24, 8.72)	0.0093	0.3000
	Age ≥ 60	Adjusted βs	Reference	6.97 (− 11.66, 25.60)	14.00 (− 3.35, 31.35)	0.0818	

Adjusted for gender, race, education level, BMI, diabetes, hypertension, cotinine, firearm noise exposure and loud noise/music exposure.

*OCPs* organochlorine pesticides, *HCB* hexachlorobenzene, *p, p'-DDE* p, p'-dichlorodiphenyldichloroethylene, *BMI* body mass index, *PTA* pure-tone average.

Significant values are in bold.

**Table 4:** Adjusted associations between OCPs and HL stratified by age (N = 366): age < 60 group (N = 308) and age ≥ 60 group (N = 58).

			Log HCB (ng/g lipid)			*P*_trend_	*P*_interaction_
			Tertile 1	Tertile 2	Tertile 3		
Low-frequency HL	Age < 60	Adjusted ORs	Reference	1.63 (0.32, 8.47)	4.38 (0.97, 19.80)	**0.0475**	**0.0101**
	Age ≥ 60	Adjusted ORs	Reference	0.81 (0.07, 9.87)	0.49 (0.03, 7.97)	0.5888	
Speech-frequency HL	Age < 60	Adjusted ORs	Reference	1.23 (0.35, 4.30)	2.16 (0.62, 7.52)	0.2316	0.4285
	Age ≥ 60	Adjusted ORs	Reference	0.54 (0.05, 5.64)	1.21 (0.10, 14.97)	0.8219	
High-frequency HL	Age < 60	Adjusted ORs	Reference	1.07 (0.54, 2.10)	1.11 (0.55, 2.24)	0.7586	0.3482
	Age ≥ 60	Adjusted ORs	Reference	6.47 (0.42, 100.64)	11.32 (0.60, 212.02)	0.1165	

			Log p, p'-DDE (ng/g lipid)			*P*_trend_	*P*_interaction_
			Tertile 1	Tertile 2	Tertile 3		
Low-frequency HL	Age < 60	Adjusted ORs	Reference	3.65 (0.56, 23.92)	16.66 (2.64, 105.09)	**0.0015**	**0.0288**
	Age ≥ 60	Adjusted ORs	Reference	0.39 (0.02, 9.67)	0.26 (0.01, 7.38)	0.4563	
Speech-frequency HL	Age < 60	Adjusted ORs	Reference	2.64 (0.65, 10.69)	7.49 (1.75, 32.01)	0.0059	0.0732
	Age ≥ 60	Adjusted ORs	Reference	0.12 (0.00, 6.34)	0.06 (0.00, 2.88)	0.1695	
High-frequency HL	Age < 60	Adjusted ORs	Reference	1.84 (0.92, 3.66)	2.30 (1.03, 5.12)	0.0357	0.3482
	Age ≥ 60	Adjusted ORs	Reference	0.15 (0.00, 10.46)	0.53 (0.01, 22.53)	0.7907	

			Log dieldrin (ng/g lipid)			*P*_trend_	*P*_interaction_
			Tertile 1	Tertile 2	Tertile 3		
Low-frequency HL	Age < 60	Adjusted ORs	Reference	6.48 (1.15, 36.46)	4.94 (0.76, 31.87)	0.0998	0.1126
	Age ≥ 60	Adjusted ORs	Reference	7.52 (0.25, 227.16)	3.66 (0.20, 66.56)	0.5247	
Speech-frequency HL	Age < 60	Adjusted ORs	Reference	3.13 (0.84, 11.66)	1.47 (0.31, 7.01)	0.6120	0.4285
	Age ≥ 60	Adjusted ORs	Reference	3.16 (0.16, 62.29)	1.19 (0.07, 19.20)	0.7664	
High-frequency HL	Age < 60	Adjusted ORs	Reference	1.25 (0.61, 2.56)	3.07 (1.44, 6.57)	0.0043	0.9242
	Age ≥ 60	Adjusted ORs	Reference	0.06 (0.00, 20.37)	0.85 (0.01, 92.86)	0.2916	

Adjusted for gender, race, education level, BMI, diabetes, hypertension, serum cotinine, firearm noise exposure and loud noise/music exposure.

*OCPs* organochlorine pesticides, *HCB* hexachlorobenzene, *p, p'-DDE* p, p'-dichlorodiphenyldichloroethylene, *BMI* body mass index, *PTA* pure-tone average, *HL* hearing loss.

Significant values are in bold.

## Discussion

In a representative sample of US adults aged 20–59 years old, higher serum HCB and p, p'-DDE concentrations were positively correlated with the prevalence of low-frequency HL and with low- and speech-frequency PTA hearing threshold shifts after adjusting for potential confounders including sex, race, education level, BMI (categorical), diabetes, hypertension, serum cotinine level, firearm noise exposure, and loud noise/music exposure. Our findings suggest that environmental exposure to OCPs may be involved in the development of hearing impairments in adults. It should be noted, however, that the 95% CI associated with the OR (16.66) of HL in the highest tertile relative to the lowest tertile of serum p, p'-DDE had a wide range (2.64–105.09).

HL is a major public health problem affecting over 1.5 billion people globally in terms of their health and quality of life^1^. The prevalence of HL has been increasing, with many factors (i.e., noise exposure, aging, and ototoxic drugs) known to be common causes, and attention has been given to the role of environmental pollutants exposure in the development of HL^2^.

Most studies on hearing impairment associated with exposure to organohalogen compounds have focused on PCBs, which are structurally related to OCPs and have similar characteristics such as being resistant to degradation, lipophilic, and able to bioaccumulate in organisms as that of OCPs^9–13^. The supporting evidence of the ototoxicity of OCPs’ is very limited, with only five studies involved. Three animal studies have investigated the ototoxicity of DDT and HCB in rats^14–16^. A study that was conducted by recording distortion product otoacoustic emissions (DPOAEs), an audiological examination performed at different ages of infants, and by calculating DPOAE amplitudes to serum OCPs reported that exposure to HCB, β-HCH, and p, p'-DDE in infancy may be linked with hearing impairment^17^. A case–control study reported a positive association between α-HCH exposure and HL in a Chinese adult population^18^. Our study is the first cross-sectional study to investigate the effect of OCPs exposure on the auditory system in a sample of the general population of US adults. The results of this study are consistent with previous findings.

Oxidative stress and/or aryl hydrocarbon receptor-mediated mechanisms may be important determinants of HCB and p, p'-DDE ototoxicity^19,20^. Aberrant epigenetic and inflammatory mechanisms caused by pesticide exposure may influence the initial stage of auditory development^21^. Although HL caused by PCBs exposure is related to the induction of hypothyroidism, and exposure to OCPs is associated with adverse thyroid function, which raises the suspicion that OCPs exposure might affect the auditory system by disturbing thyroid function; however, no evidence has supported this hypothesis^14,22,23^. In addition, whether exposure to HCB and p, p'-DDE causes ototoxicity by affecting the cochlear outer hair cells (OHCs) or other parts of the auditory system (i.e., the organ of Corti, the nerves) remains uncertain: one study showed that DPOAEs (measure for assessing OHCs function) was affected by HCB and p, p'-DDE exposure, and another study observed only a slight loss of hair cells (< 1%) in HCB-treated rats, which could also be observed in control animals^14,17^. In addition, results regarding the range of hearing impairment caused by exposure to OCPs have been inconsistent across studies. In our study, HCB and p, p'-DDE exposure was positively associated with low-frequency HL and with low- and speech-frequency hearing threshold shifts. These results are almost in agreement with those of the study by Hadjab et al.^14^. Contrarily, Sisto et al.^17^ found positive associations between HCB and p, p'-DDE exposure and DPOAE amplitudes for most of the primary tone frequencies. Zhang et al.^18^ observed a relationship between α-HCH exposure and hearing impairment, mainly at mid and high frequencies. Further studies are required to clarify these discrepancies.

This study used a large and representative sample with rigorous quality control over the data collection process and adjusted for critical confounders to establish a relationship between serum OCPs concentrations and hearing impairment. However, this study had several limitations. First, due to the inherent limitation of the cross-sectional design, the temporal sequence of exposures and outcomes cannot be confirmed. Prospective studies are required to definitively define the association between OCPs exposure and hearing function. Second, data of infants, adolescents, and the elderly (70 + years old) were not included in this study due to the lack of participants or the sample size being too small. Moreover, we could not rule out any potential effects of OCPs exposure on hearing impairment in people aged ≥ 60 years, as the sample size in the subgroup was small (N = 58). Third, some potential confounders (e.g., dietary intake, history of ear infection, and congenital hearing impairment) were not addressed in this study. Furthermore, studies on the mechanisms of the effects of HCB and p, p′-DDE exposure on hearing impairment are insufficient. Therefore, functional biological studies (i.e., DPOAE and brainstem auditory evoked potential studies) and prospective population studies are required to investigate the potential mechanisms.

## Methods

### Study design and population

The NHANES is a nationwide, cross-sectional, representative series of surveys containing health-related information of the US general population. These continuous surveys consist of interviews, physical measurements, and laboratory tests of a selected sample of the US non-institutionalized population. The NHANES project was reviewed and approved by the Research Ethics Review Board of the National Center for Health Statistics, and informed consent was obtained from all participants. Data from the NHANES and related documentation and protocols described in detail are publicly available from the NHANES website (https://www.cdc.gov/nchs/nhanes/Index.htm).

**Figure 1:**
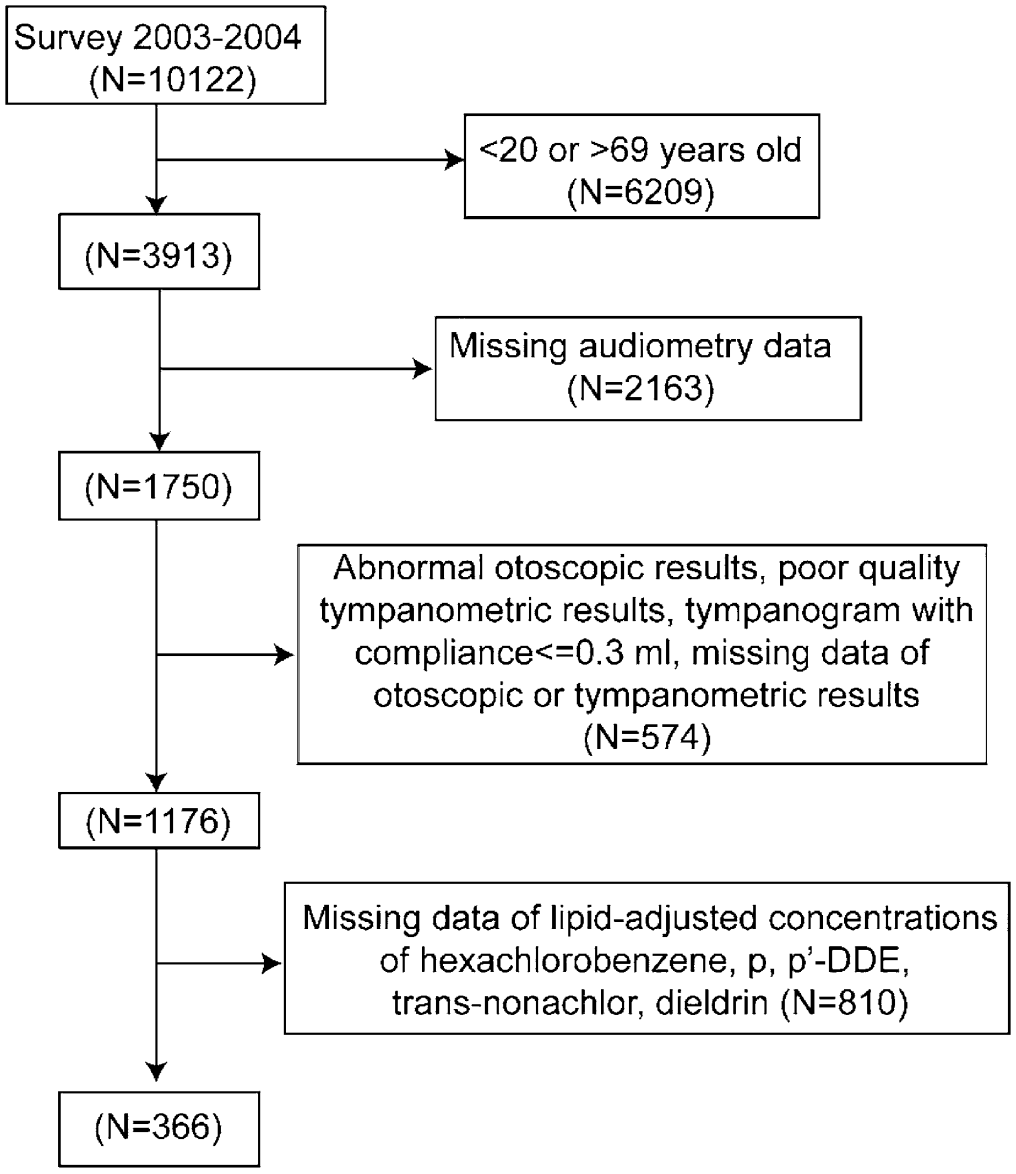
Flow chart of participant selection. Abbreviations: p, p'-DDE, p, p'-dichlorodiphenyldichloroethylene.

The participants in this report were enrolled from the NHANES cycle 2003–2004, which contains the results of both serum concentrations of OCPs and audiometry examinations of 20–69-year-old adults. Since the analytical detection limits of serum OCPs varied significantly in previous cycles and the sample sizes of recent cycles were quite small, only the data from the 2003–2004 cycle were used in our analysis. Figure 1 shows a flow chart for participant selection in this study. Participants with missing data on hearing threshold levels, otoscopic tests, tympanogram tests, or serum lipid-adjusted concentrations of the selected OCPs were excluded. Individuals with abnormal otoscopic results, poor-quality tympanogram results, or tympanograms with compliance ≤ 0.3 ml were also excluded to avoid analyzing conductive or mixed HL data^24,25^. Finally, 366 participants were included in this study.

### OCPs exposure measurement

Blood serum concentrations of 13 OCPs and metabolites were measured in a random one-third subsample of participants aged 12 years and older using high-resolution gas chromatography/isotope-dilution high-resolution mass spectrometry at the US Centers for Disease Control and Prevention (CDC) National Center for Environmental Health. Four OCPs detected in ≥ 80% of the samples were selected for analysis in our study: HCB, p, p'-DDE, trans-nonachlor, and dieldrin. We used the limit of detection (LOD) divided by the square root of two for any sample below the LOD. In addition, the lipid-adjusted serum concentrations (ng/g lipid) of the four OCPs were used in our study and log-transformed to normalize the skewed distribution before the analysis.

### Audiometric measurement

The detailed procedure and protocol of audiometric examination have been described in the NHANES audiometry procedures online manual^26^ and previous papers^27,28^. Briefly, half of the sample of US adults aged 20–69 years underwent an audiometric component test. Trained examiners conducted hearing threshold examinations in a silent and sound-isolating audiometry room. The hearing threshold examination was conducted at seven frequencies from 500 to 8000 Hz using an AD226 audiometer (Interacoustics). In this study, low-frequency HL was defined as PTA calculated by averaging thresholds at 500, 1000, and 2000 Hz ≥ 20 dB HL in the better ear; speech-frequency HL was defined as PTA at 500, 1000, 2000, and 4000 Hz ≥ 20 dB HL in the better ear, which is consistent with the definition used by the World Health Organization^1^; and high-frequency HL was defined as PTA at 4000, 6000, and 8000 Hz ≥ 20 dB HL in the better ear.

Otoscopic examination of both ears was performed using a Welch Allyn otoscope (Model 25,020). Tympanometry was performed using an Earscan Acoustic Impedance tympanometer (Micro Audiometrics) to assess middle ear function. Inference of sensorineural HL was based on the findings of normal otoscopic examinations and good- or adequate-quality results of the tympanogram with compliance > 0.3 ml. Individuals who did not meet the standards were excluded from further analysis.

### Covariates

The following variables were considered as potential covariates in the analysis: age and BMI as continuous variables, and sex, race/ethnicity, education level, BMI (categorical), diabetes, hypertension, serum cotinine level, firearm noise exposure, and noise noise/music exposure as categorical variables. Information on demographic variables, noise exposure, and present medical illnesses, such as diabetes and hypertension, was obtained from self-reported questionnaires. Firearm exposure was defined as “firearm noise exposure outside work for an average of at least once a month for a year”^29^. Loud noise/music exposure was defined as “exposure to other types of loud noise, such as noise from power tools or loud music, outside work, for an average of at least once a month for a year.” Diabetes was defined by a “yes” or “borderline” answer to the question “other than during pregnancy, ever been told by a doctor or health professional had diabetes or sugar diabetes”^30^. Hypertension was defined as “ever been told by a doctor or other health professional had hypertension, also called high blood pressure”^30^. BMI was obtained through physical examination. Serum cotinine level, a marker for both active and passive tobacco exposure, was tested by an isotope dilution-high-performance liquid chromatography/atmospheric pressure chemical ionization tandem mass spectrometry^31^.

### Statistical analysis

Weighted statistical differences in demographic and potential hearing-related variables between individuals grouped by sex were evaluated, with categorical data presented as percentages and continuous data as mean ± SD. The *P* values of the continuous and categorical data were estimated using a weighted linear regression model and weighted chi-square test, respectively. We distributed the log-transformed lipid-adjusted OCP levels into tertiles before conducting univariate analysis to estimate potential variables. Multivariate linear regression analysis was used to determine regression coefficients (β) and 95% CIs between the four OCPs and hearing threshold shifts, and multivariate logistic regression analysis was used to estimate ORs and 95% CIs between the four OCPs and HL, adjusting for age, gender, race/ethnicity, education level, BMI (categorical), diabetes, hypertension, serum cotinine level, firearm noise exposure, and loud noise/music exposure, instead of using sample weights. This adjustment is considered a good compromise between efficiency and bias^32,33^. We evaluated the influence of the interactions between the OCPs and age on HL. Stratified multivariate linear and logistic regression analyses according to age (< 60 vs. ≥ 60 years) were performed. The association between trans-nonachlor exposure and HL was not estimated in the multivariate linear and logistic regression analyses stratified by age because the number of participants aged ≥ 60 years in the category of low trans-nonachlor exposure tertile was quite small, and only two individuals were included. Statistical analysis was conducted using the statistical programming language R 3.6.1 and EmpowerStats software (X&Y Solutions, Inc.). A *P*-value < 0.05 was considered statistically.

## Supplementary Information

Supplementary Information.
