# Supplementary material for: Exploring the association of organochlorine pesticides exposure and hearing impairment in United States adults
Source: Sci Rep. 2022 Jul 13;12:11887. doi: 10.1038/s41598-022-15892-2 (PMC9279322; doi:10.1038/s41598-022-15892-2)
Supplement: Supplementary file 1 — Supplementary Information. [file 41598_2022_15892_MOESM1_ESM.docx]

|  | | Log HCB (ng/g lipid) | | | *P*_trend_ |
| --- | --- | --- | --- | --- | --- |
|  |  | Tertile 1 | Tertile 2 | Tertile 3 |  |
| Low-frequency PTA | Crude βs | Reference | 2.09 (0.17, 4.00) | 2.54 (0.64, 4.45) | 0.0093 |
|  | Adjusted βs | Reference | 0.21 (-1.50, 1.92) | 0.16 (-1.57, 1.90) | 0.8551 |
| Speech-frequency PTA | Crude βs | Reference | 2.73 (0.59, 4.87) | 2.84 (0.71, 4.98) | 0.0096 |
|  | Adjusted βs | Reference | 0.12 (-1.63, 1.86) | 0.06 (-1.72, 1.83) | 0.9508 |
| High-frequency PTA | Crude βs | Reference | 3.81 (-0.28, 7.89) | 4.33 (0.26, 8.40) | 0.0378 |
|  | Adjusted βs | Reference | -1.48 (-4.63, 1.66) | -1.26 (-4.45, 1.93) | 0.4391 |
|  | | Log p, p'-DDE (ng/g lipid) | | | *P*_trend_ |
|  |  | Tertile 1 | Tertile 2 | Tertile 3 |  |
| Low-frequency PTA | Crude βs | Reference | 1.31 (-0.58, 3.20) | 3.77 (1.88, 5.66) | 0.0001 |
|  | Adjusted βs | Reference | -0.49 (-2.25, 1.26) | -1.01 (-3.11, 1.10) | 0.3480 |
| Speech-frequency PTA | Crude βs | Reference | 1.38 (-0.72, 3.48) | 4.93 (2.83, 7.03) | <0.0001 |
|  | Adjusted βs | Reference | -1.03 (-2.82, 0.76) | -1.06 (-3.21, 1.09) | 0.3206 |
| High-frequency PTA | Crude βs | Reference | 3.16 (-0.85, 7.16) | 8.76 (4.75, 12.76) | <0.0001 |
|  | Adjusted βs | Reference | -2.07 (-5.28, 1.15) | -3.64 (-7.50, 0.22) | 0.0636 |
|  |  | Log trans-nonachlor (ng/g lipid) | | | *P*_trend_ |
|  |  | Tertile 1 | Tertile 2 | Tertile 3 |  |
| Low-frequency PTA | Crude βs | Reference | 3.37 (1.55, 5.20) | 6.05 (4.23, 7.88) | <0.0001 |
|  | Adjusted βs | Reference | 0.45 (-1.35, 2.25) | -0.60 (-2.85, 1.65) | 0.6224 |
| Speech-frequency PTA | Crude βs | Reference | 3.84 (1.82, 5.86) | 7.54 (5.52, 9.56) | <0.0001 |
|  | Adjusted βs | Reference | 0.00 (-1.83, 1.84) | -1.32 (-3.61, 0.97) | 0.2705 |
| High-frequency PTA | Crude βs | Reference | 6.86 (3.03, 10.69) | 14.40 (10.57, 18.23) | <0.0001 |
|  | Adjusted βs | Reference | -0.58 (-3.89, 2.73) | -2.77 (-6.90, 1.36) | 0.1951 |
|  | | Log dieldrin (ng/g lipid) | | | *P*_trend_ |
|  |  | Tertile 1 | Tertile 2 | Tertile 3 |  |
| Low-frequency PTA | Crude βs | Reference | 2.72 (0.83, 4.62) | 3.82 (1.95, 5.70) | <0.0001 |
|  | Adjusted βs | Reference | 1.41 (-0.31, 3.14) | 0.24 (-1.68, 2.16) | 0.7611 |
| Speech-frequency PTA | Crude βs | Reference | 2.79 (0.67, 4.90) | 5.12 (3.03, 7.21) | <0.0001 |
|  | Adjusted βs | Reference | 0.95 (-0.81, 2.71) | 0.16 (-1.81, 2.12) | 0.8461 |
| High-frequency PTA | Crude βs | Reference | 4.31 (0.35, 8.26) | 11.29 (7.38, 15.21) | <0.0001 |
|  | Adjusted βs | Reference | 0.96 (-2.22, 4.14) | 1.59 (-1.95, 5.14) | 0.3755 |

Table S1. Adjusted^a^ associations between OCPs and hearing threshold shifts (N=366). Abbreviations: OCPs, organochlorine pesticides; HCB, hexachlorobenzene; p, p'-DDE, p, p'-dichlorodiphenyldichloroethylene; BMI, body mass index; PTA, pure-tone average. ^a^ Adjusted for age, gender, race/ethnicity, education level, BMI (categorical), diabetes, hypertension, serum cotinine, firearm noise exposure, and loud noise/music exposure.

|  | | Log HCB (ng/g lipid) | | | *P*_trend_ |
| --- | --- | --- | --- | --- | --- |
|  |  | Tertile 1 | Tertile 2 | Tertile 3 |  |
| Low-frequency HL | Crude ORs | Reference | 1.93 (0.69, 5.41) | 2.48 (0.92, 6.69) | 0.0730 |
|  | Adjusted ORs | Reference | 1.03 (0.30, 3.54) | 1.56 (0.45, 5.45) | 0.4524 |
| Speech-frequency HL | Crude ORs | Reference | 1.54 (0.68, 3.47) | 2.57 (1.20, 5.50) | 0.0124 |
|  | Adjusted ORs | Reference | 0.67 (0.23, 1.90) | 1.27 (0.45, 3.59) | 0.5826 |
| High-frequency HL | Crude ORs | Reference | 1.47 (0.86, 2.50) | 1.64 (0.96, 2.78) | 0.0703 |
|  | Adjusted ORs | Reference | 0.70 (0.33, 1.49) | 0.77 (0.36, 1.67) | 0.5184 |
|  | | Log p, p'-DDE (ng/g lipid) | | | *P*_trend_ |
|  |  | Tertile 1 | Tertile 2 | Tertile 3 |  |
| Low-frequency HL | Crude ORs | Reference | 1.80 (0.51, 6.30) | 5.78 (1.91, 17.48) | 0.0005 |
|  | Adjusted ORs | Reference | 1.09 (0.25, 4.72) | 2.30 (0.53, 9.94) | 0.1894 |
| Speech-frequency HL | Crude ORs | Reference | 1.96 (0.75, 5.09) | 5.84 (2.46, 13.85) | <0.0001 |
|  | Adjusted ORs | Reference | 0.95 (0.29, 3.11) | 1.34 (0.38, 4.69) | 0.5720 |
| High-frequency HL | Crude ORs | Reference | 1.55 (0.89, 2.71) | 3.07 (1.78, 5.28) | <0.0001 |
|  | Adjusted ORs | Reference | 0.79 (0.35, 1.75) | 0.63 (0.25, 1.58) | 0.3292 |
|  |  | Log trans-nonachlor (ng/g lipid) | | | *P*_trend_ |
|  |  | Tertile 1 | Tertile 2 | Tertile 3 |  |
| Low-frequency HL | Crude ORs | Reference | 3.54 (0.95, 13.20) | 6.87 (1.97, 23.97) | 0.0011 |
|  | Adjusted ORs | Reference | 1.08 (0.23, 5.14) | 0.64 (0.11, 3.55) | 0.4377 |
| Speech-frequency HL | Crude ORs | Reference | 3.03 (1.06, 8.70) | 8.68 (3.26, 23.12) | <0.0001 |
|  | Adjusted ORs | Reference | 0.67 (0.18, 2.56) | 0.48 (0.11, 2.03) | 0.3016 |
| High-frequency HL | Crude ORs | Reference | 2.49 (1.35, 4.58) | 7.34 (4.03, 13.38) | <0.0001 |
|  | Adjusted ORs | Reference | 0.97 (0.43, 2.21) | 0.87 (0.34, 2.21) | 0.7519 |
|  | | Log dieldrin (ng/g lipid) | | | *P*_trend_ |
|  |  | Tertile 1 | Tertile 2 | Tertile 3 |  |
| Low-frequency HL | Crude ORs | Reference | 3.25 (1.02, 10.38) | 3.99 (1.28, 12.39) | 0.0172 |
|  | Adjusted ORs | Reference | 3.08 (0.81, 11.76) | 1.63 (0.38, 7.02) | 0.6725 |
| Speech-frequency HL | Crude ORs | Reference | 3.06 (1.24, 7.59) | 4.28 (1.78, 10.28) | 0.0010 |
|  | Adjusted ORs | Reference | 1.88 (0.59, 6.00) | 0.57 (0.15, 2.09) | 0.2129 |
| High-frequency HL | Crude ORs | Reference | 1.55 (0.87, 2.76) | 4.15 (2.39, 7.23) | <0.0001 |
|  | Adjusted ORs | Reference | 0.92 (0.42, 2.02) | 1.53 (0.67, 3.51) | 0.2987 |

Table S2. Adjusted^a^ associations between OCPs and HL (N=366).Abbreviations: OCPs, organochlorine pesticides; HCB, hexachlorobenzene; p, p'-DDE, p, p'-dichlorodiphenyldichloroethylene; BMI, body mass index; PTA, pure-tone average; HL, hearing loss.^a^ Adjusted for age, gender, race/ethnicity, education level, BMI (categorical), diabetes, hypertension, serum cotinine, firearm noise exposure and loud noise/music exposure.
